# Supplementary material for: Analysis of Plant–Fungus Interactions in Calocybe gambosa Fairy Rings
Source: Plants (Basel). 2025 Sep 17;14(18):2884. doi: 10.3390/plants14182884 (PMC12473715; doi:10.3390/plants14182884)
Supplement: Supplementary file 1 [file plants-14-02884-s001.zip › plants-3837547-supplementary.pdf]

Analysis of Plant–Fungus Interactions in *Calocybe gambosa* Fairy Rings

Simone Graziosi[1]*, Alessandra Lombini[1], Federico Puliga[1], Hillary Righini[1], Ludovico Dalla Pozza[1], Veronica Zuffi[1], Mirco Iotti[2], Ornella Francioso[1], Roberta Roberti[1], Alessandra Zambonelli[1]

[1]Department of Agricultural and Food Sciences, University of Bologna, Viale G. Fanin 40-44, 40127, Bologna, Italy; [2]Department of Life, Health and Environmental Science, University of L'Aquila, Via Vetoio, 67100, Coppito, L'Aquila, Italy;

*Corresponding author: simone.graziosi5@unibo.it

Co-author details:

Alessandra Lombini, Department of Agricultural and Food Sciences, University of Bologna, Viale G. Fanin 44, 40127, Bologna, Italy, email: alessandra.lombini@unibo.it
Alessandra Zambonelli, Department of Agricultural and Food Sciences, University of Bologna, Viale G. Fanin 44, 40127, Bologna, Italy, email: alessandr.zambonelli@unibo.it
Federico Puliga, Department of Agricultural and Food Sciences, University of Bologna, Viale G. Fanin 44, 40127, Bologna, Italy, email: federico.puliga2@unibo.it
Hillary Righini, Department of Agricultural and Food Sciences, University of Bologna, Viale G. Fanin 40, 40127, Bologna, Italy, email: hillary3001@gmail.com
Ludovico dalla Pozza, Department of Agricultural and Food Sciences, University of Bologna, Viale G. Fanin 44, 40127, Bologna, Italy, email: ludovico.dallapozza@studio.unibo.it
Mirco Iotti, Department of Life, Health and Environmental Science, University of L'Aquila, Via Vetoio, 67100, Coppito, L'Aquila, Italy, email: mirco.iotti@univaq.it
Ornella Francioso, Department of Agricultural and Food Sciences, University of Bologna, Viale G. Fanin 40, 40127, Bologna, Italy, email: ornella.francioso@unibo.it
Roberta Roberti, Department of Agricultural and Food Sciences, University of Bologna, Viale G. Fanin 40, 40127, Bologna, Italy, email: roberta.roberti@unibo.it
Veronica Zuffi, Department of Agricultural and Food Sciences, University of Bologna, Viale G. Fanin 40, 40127, Bologna, Italy, email: veronica.zuffi3@unibo.it

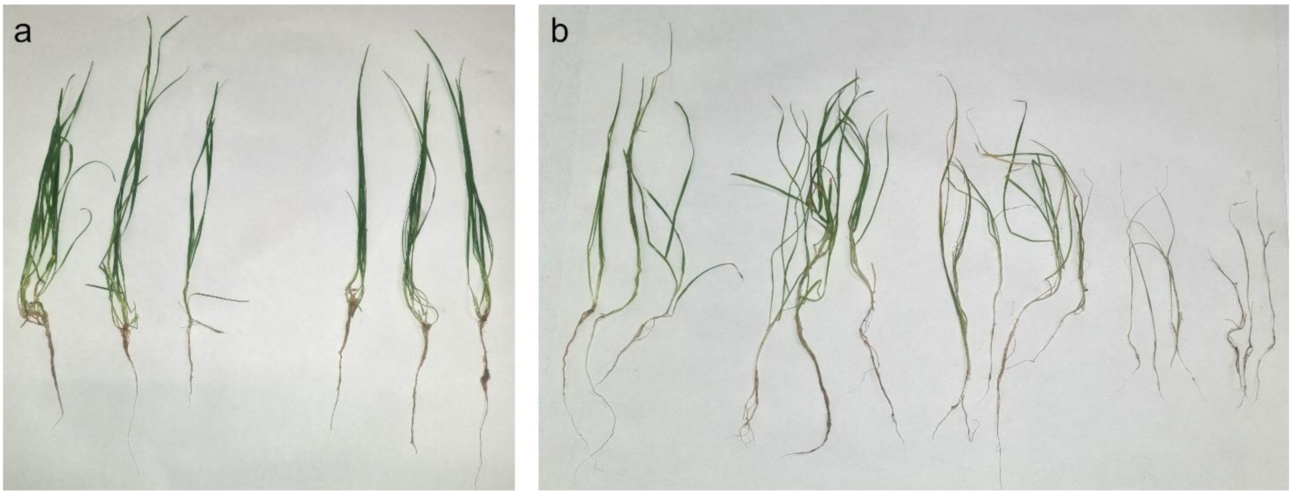

Figure S 1. Damage categories of *Poa trivialis* L. plants after three months of *ex situ* co-culture experiments with *Calocybe gambosa* under semi-natural conditions. (a) Plant samples were grouped and categorized based on damage categories and collected from control vessels without mycelium presence (b) and test vessels with co-occurring mycelium.

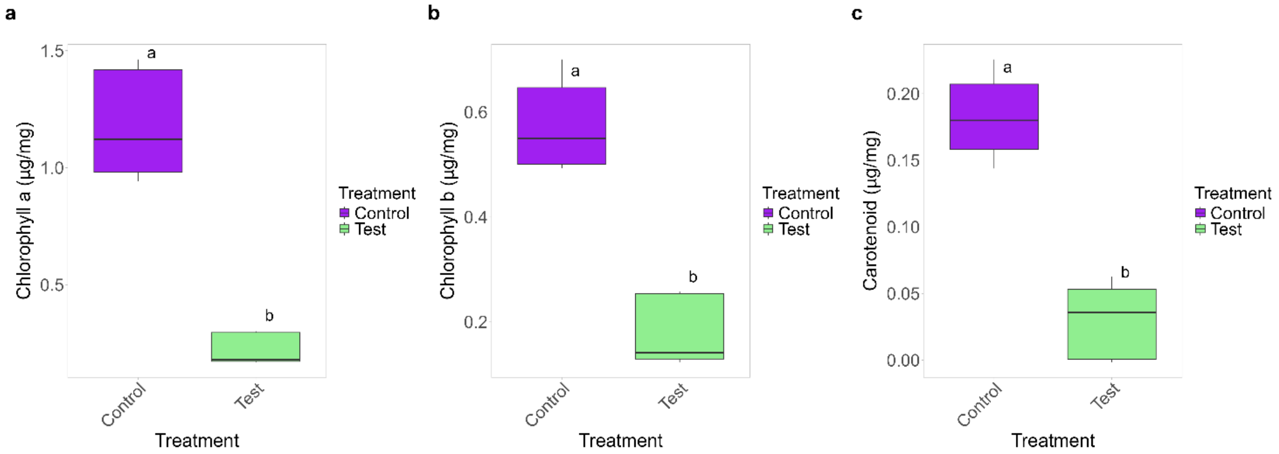

Figure S 2. Chlorophyll-a,-b, and carotenoid determination was performed *ex situ* on *Poa trivialis* plants cultivated in the presence of *Calocybe gambosa* mycelium: (a) chlorophyll a; (b) chlorophyll b; carotenoid (c). Different letters indicate a difference between treatments according to Tukey's test ($p < 0.05$).

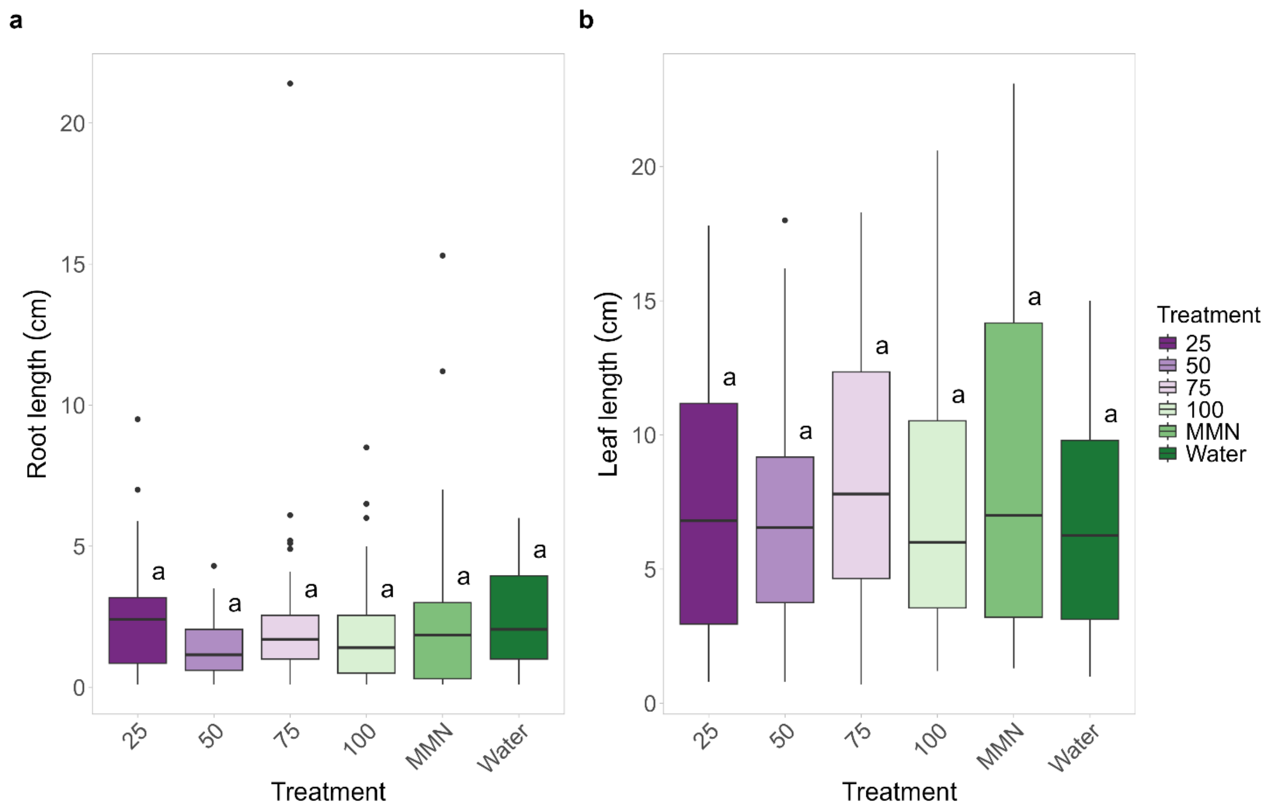

Figure S 3. *Ex situ* effects on *Poa trivialis* root (a) and leaf (b) elongation of increasing concentration addition (25%, 50%, 75%, 100%) of *Calocybe gambosa* liquid media after mycelial cultivation and controls (MMN, Water). Different letters indicate a difference between treatments according to Tukey's test ($p < 0.05$).

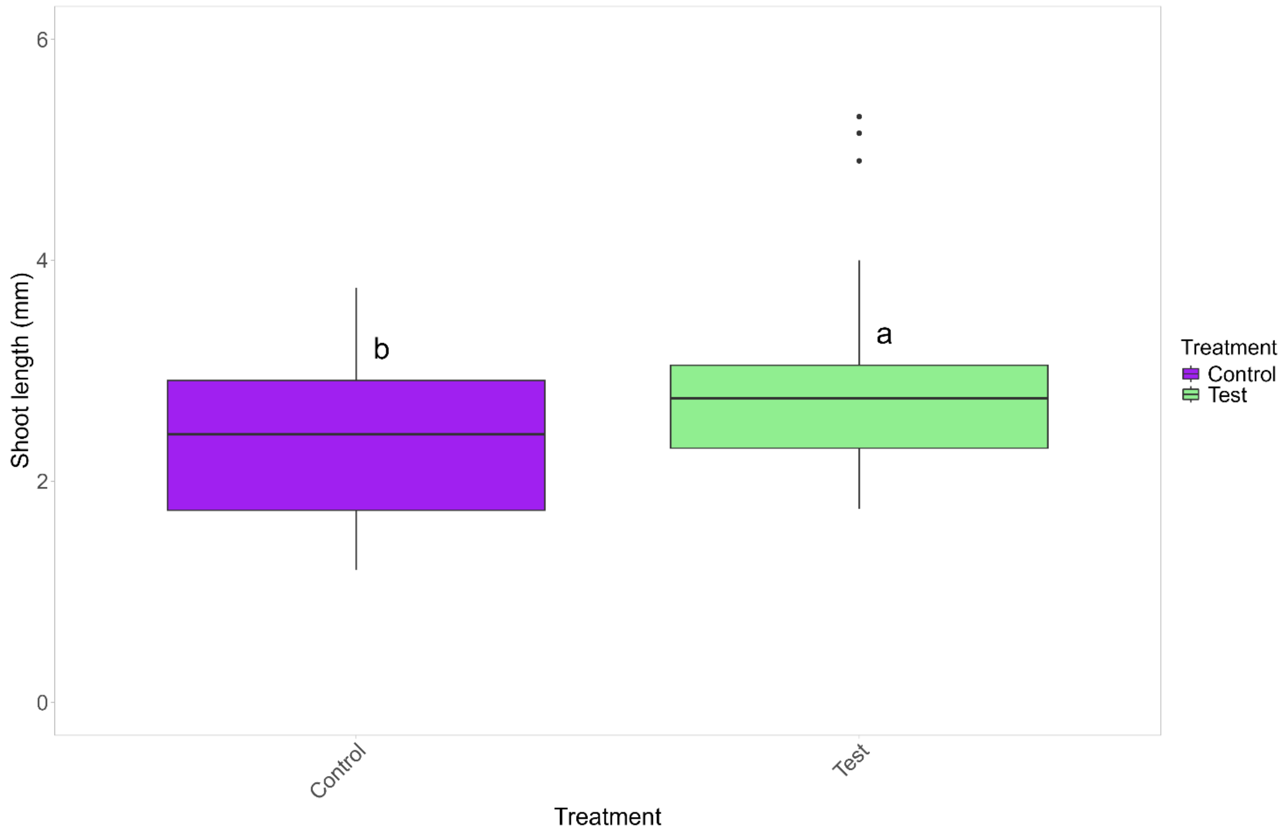

Figure S 4. *Ex situ* influence of volatile organic compounds (VOCs) produced by *Calocybe gambosa* mycelium on *Poa trivialis* shoot elongation after a co-culture experiment in an insulated system. Different letters indicate a difference between treatments according to Tukey's test ($p < 0.05$).

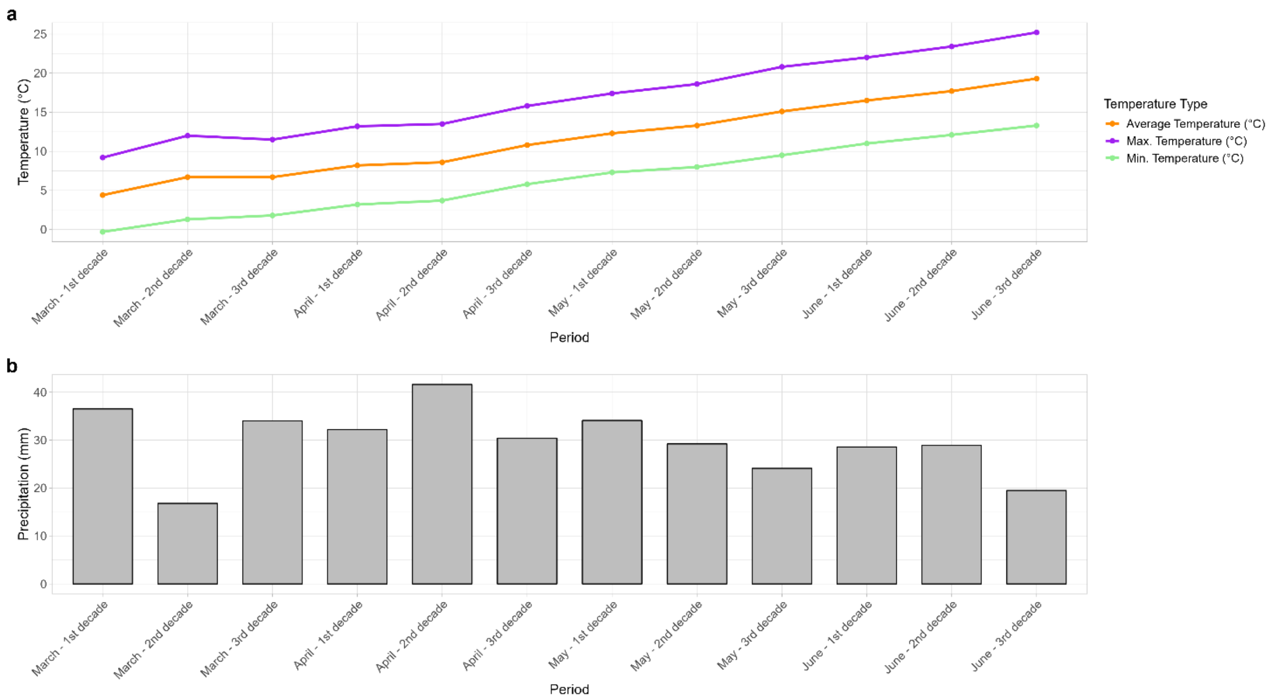

Figure S 5. Climatological data (1991–2020) during the *Calocybe gambosa* fructification period (March–June). (a) Minimum, maximum, and average temperatures (°C) over successive 10-day periods; (b) average precipitation (mm) over the same intervals.

Table S 1. List of plant species collected in the areas of fairy rings (FRS): external area (EX), fungal front (FF), greener belt (GB), IN (internal area). For each species is reported the respective family, life form, and the morphological type.

Nr.	Species	IN[a]	GB[a]	FF[a]	EX[a]	Family	Morphological Type	Life Form
**1**	*Ajuga reptans* L.				x	Lamiaceae	Dicotyledon	Perennial
**2**	*Anthoxanthum odoratum* L.	x	x	x	x	Poaceae	Monocotyledon	Perennial
**3**	*Bellis perennis* L.	x	x	x		Asteraceae	Dicotyledon	Perennial
**4**	*Cirsium arvense* (L.) Scop.				x	Asteraceae	Dicotyledon	Perennial
**5**	*Cruciata glabra* (L.) C.Bauhin				x	Rubiaceae	Dicotyledon	Perennial
**6**	*Dactylis glomerata* L.	x	x	x	x	Poaceae	Monocotyledon	Perennial
**7**	*Dacus carota* L.	x	x	x	x	Apiaceae	Dicotyledon	Annual-Biennial
**8**	*Festuca rubra* L.	x	x	x	x	Poaceae	Monocotyledon	Perennial
**9**	*Galium verum* L.		x		x	Rubiaceae	Dicotyledon	Perennial
**10**	*Holcus lanatus* L.	x	x	x	x	Poaceae	Monocotyledon	Perennial
**11**	*Lathyrus pratensis* L.				x	Fabaceae	Dicotyledon	Perennial
**12**	*Leuchanthemum vulgare*				x	Asteraceae	Dicotyledon	Perennial
**13**	*Lolium perenne* L.	x	x	x		Poaceae	Monocotyledon	Perennial
**14**	*Lychnis flos-cuculi* L.		x		x	Caryophyllaceae	Dicotyledon	Perennial
**15**	*Myosotis arvensis* (L.) Hill		x		x	Boraginaceae	Dicotyledon	Annual-Biennial
**16**	*Pastinaca sativa* L.	x	x	x	x	Apiaceae	Dicotyledon	Annual-Biennial
**17**	*Pimpinella saxifraga* L.	x	x	x	x	Apiaceae	Dicotyledon	Perennial
**18**	*Plantago lanceolata* L.			x	x	Plantaginaceae	Dicotyledon	Perennial
**19**	*Poa sylvicola* Guss.	x	x	x	x	Poaceae	Monocotyledon	Perennial
**20**	*Poa trivialis* L.	x	x	x		Poaceae	Monocotyledon	Perennial
**21**	*Ranunculus bulbosus* L.	x	x	x	x	Ranunculaceae	Dicotyledon	Perennial
**22**	*Rumex acetosa* L.	x	x	x	x	Polygonaceae	Dicotyledon	Perennial
**23**	*Salvia prantesis* L.				x	Lamiaceae	Dicotyledon	Perennial
**24**	*Taraxacum* sp.	x	x	x	x	Asteraceae	Dicotyledon	Perennial
**25**	*Tragopogon pratensis* L.				x	Asteraceae	Dicotyledon	Annual-Biennial
**26**	*Trifolium pratense* L.	x	x	x	x	Fabaceae	Dicotyledon	Perennial
**27**	*Veronica chamaedrys* L.				x	Plantaginaceae	Dicotyledon	Perennial
**28**	*Vicia sativa* L.				x	Fabaceae	Dicotyledon	Annual-Biennial

Note: a = In the table, a single detection of a species in a zone (IN, GB, FF, EX) within one of the three FRs (FR1, FR2, FR3) is sufficient to register its overall presence in that FR area.

Table S 2. Enzymatic plate assay to determine the production of enzymes related to pathogenic lifestyle: endo-1,4-*β*-glucanase, polygalacturonase, polymethylgalacturonase, and xylanase.

**Enzyme**	**Halo area (cm^2)**	**Mycelial area (cm^2)**
Endo-1,4-*β*-glucanase	4.27 ± 1.09	3.32 ± 0.14
Polygalacturonase	1.49 ± 0.18	3.06 ± 0.39
Polymethylgalacturonase	2.21 ± 0.36	7.65 ± 0.89
Xylanase	1.71 ± 0.62	14.80 ± 1.80

Note: The halo area and mycelial area are reported as mean ± standard error (SE).

Table S 3. Fairy rings (FRs) of *Calocybe gambosa* involved in this study at the Montefiorino (Modena, Italy) sampling site. For each FR, GPS coordinates, altitude, average radius, and location were recorded.

**Fairy ring (FR)**	**GPS coordinates**	**Altitude (a.s.l. m)**	**Representative FR plant species**	**Average radius (m)**	**Internal area (m^2)**	**Fungal front area (m^2)**	**Greener belt area (m^2)**	**External area (m^2)**
1	44.3465783, 10.6093973	849	*Anthoxanthum odoratum* L., *Dactylis glomerata* L., *Festuca rubra* L.	3.00 ± 0.003	3.14 ± 0.18	1.47 ± 0.045	1.28 ± 0.045	7.85 ± 0.18
2	44.3468135, 10.6095130	845	*Anthoxanthum odoratum* L., *Dactylis glomerata* L., *Holcus lanatus* L.	3.50 ± 0.32	4.71 ± 1.75	1.87 ± 0.44	1.67 ± 0.44	9.42 ± 1.75
3	44.3461199, 10.6096313	863	*Dactylis glomerata L., Festuca rubra* L.	2.87 ± 0.096	2.72 ± 0.52	1.37 ± 0.13	1.17 ± 0.52	7.44 ± 0.52

Note: The average radius, internal area, fungal front area, greener belt area, and external area are reported as mean ± standard error (SE).

Table S 4. List of *Calocybe gambosa* strains isolated in this study and used in the *C. gambosa* species-specific primer test.

Strain	NCBI ITS (rDNA) accession number	Habitat	Main vegetal species	Localization[a]	Date
Calgam0	NA	Broadleaf mixed forest	*Acer campestre* L. *Crataegus monogyna* Jacq. *Prunus spinosa* L. *Quercus* spp.	Pavullo (MO), Emilia Romagna, Italy	27/04/2021
Calgam1	PV628678	Broadleaf mixed forest	*Crataegus monogyna* Jacq. *Prunus spinosa* L. *Quercus* spp.	Valsamoggia (BO), Emilia Romagna, Italy	02/05/2022
Calgam2	PV628679	Broadleaf mixed forest	*Crataegus monogyna* Jacq. *Prunus spinosa* L. *Quercus* spp.	Valsamoggia (BO), Emilia Romagna, Italy	25/04/2022
Calgam3	PV628680	Broadleaf mixed forest	*Crataegus monogyna* Jacq. *Prunus spinosa* L. *Quercus* spp.	Valsamoggia (BO), Emilia Romagna, Italy	26/04/2022
Calgam4	PV628681	Broadleaf mixed forest	*Crataegus monogyna* Jacq. *Prunus spinosa* L. *Quercus* spp.	Valsamoggia (BO), Emilia Romagna, Italy	28/04/2022
Calgam5	PV628682	Broadleaf mixed forest	*Crataegus monogyna* Jacq. *Prunus spinosa* L. *Quercus* spp.	Valsamoggia (BO), Emilia Romagna, Italy	02/05/2022
Calgam6	PV628683	Broadleaf mixed forest	*Crataegus monogyna* Jacq. *Prunus spinosa* L. *Quercus* spp.	Valsamoggia (BO), Emilia Romagna, Italy	02/05/2022

Calgam7	PV628684	Broadleaf mixed forest	*Crataegus monogyna* Jacq. *Prunus spinosa* L. *Quercus* spp.	Valsamoggia (BO), Emilia Romagna, Italy	02/05/2022
Calgam8	PV628685	Broadleaf mixed forest	*Crataegus monogyna* Jacq. *Fraxinus ornus* L. *Prunus spinosa* L. *Quercus* spp.	Premilcuore (FC), Emilia Romagna, Italy	09/05/2022
Calgam9	PV628686	Broadleaf mixed forest	*Crataegus monogyna* Jacq. *Fraxinus ornus* L. *Prunus spinosa* L. *Quercus* spp.	Premilcuore (FC), Emilia Romagna, Italy	09/05/2022
Calgam10	PV628687	Broadleaf mixed forest	*Crataegus monogyna* Jacq. *Prunus spinosa* L. *Quercus* spp.	Valsamoggia (BO), Emilia Romagna, Italy	10/05/2022
Calgam11	NA	Broadleaf mixed forest	*Crataegus monogyna* Jacq. *Prunus spinosa* L. *Quercus* spp.	Valsamoggia (BO), Emilia Romagna, Italy	09/06/2022
Calgam12	PV628688	Grassland	Predominance of Poaceae	Montefiorino (MO), Emilia Romagna, Italy	21/04/2023
Calgam13	PV628689	Broadleaf mixed forest	*Crataegus monogyna* Jacq. *Prunus spinosa* L. *Quercus* spp.	Castelvetro (MO), Emilia Romagna, Italy	21/04/2023
Calgam14	PV628690	Broadleaf mixed forest	*Crataegus monogyna* Jacq. *Prunus spinosa* L. *Quercus* spp.	Valsamoggia (BO), Emilia Romagna, Italy	02/04/2024

Calgam15	PV628691	Broadleaf mixed forest	*Crataegus monogyna* Jacq. *Prunus spinosa* L. *Quercus* spp.	Valsamoggia (BO), Emilia Romagna, Italy	02/04/2024
Calgam16	PV628692	Broadleaf mixed forest	*Crataegus monogyna* Jacq. *Prunus spinosa* L. *Quercus* spp.	Valsamoggia (BO), Emilia Romagna, Italy	02/04/2024
Calgam17	PV628693	Broadleaf mixed forest	*Acer campestre* L. *Crataegus monogyna* Jacq. *Prunus spinosa* L. *Quercus* spp.	Sestola (MO), Emilia Romagna, Italy	15/04/2024
Calgam18	PV628694	Grassaland	Predominance of Poaceae	Montefiorino (MO), Emilia Romagna, Italy	17/04/2024
Calgam19	PV628695	Grassland	Predominance of Poaceae	Montefiorino (MO), Emilia Romagna, Italy	17/04/2024
Calgam20	PV628696	Grassland	Predominance of Poaceae	Montefiorino (MO), Emilia Romagna, Italy	17/04/2024

Note: [a]BO= Bologna, MO = Modena, FC = Forli-Cesena;

Table S 5. Oligonucleotide primers and PCR cycling conditions used in this study.

**Primer**	**Sequence (5' – 3')**	**Target gene**	**PCR cycling conditions**	**Reference**
ITS1f	CTTGGTCATTTAGAGGAAGTAA	ITS rRNA	95 °C 6 min; 34 x (94 °C 30 s, 56 °C 30 s, 72 °C 1 min), 72 °C 7 min	[89,90]
ITS4	TCCTCCGCTTATTGATATGC	ITS rRNA		
CalgamI	TGGAGGATTTGCAATGGTGTG	ITS rRNA	94 °C 5 min; 25 x (94 °C 20 s, 62 °C 15 s, 72 °C 30 sec), 72 °C 7 min	This study
CalgamII	GCCGACACAACAGTTAGAAGC	ITS rRNA		

Table S 6. ITS (rDNA) sequences employed for *Calocybe gambosa* PCR species-specific primer design. These sequences were obtained from fungal genomic data published in NCBI GenBank [92].

Species	Order	Family	Accession number NCBI	Geographical origin[a]
Agaricus bisporus (J.E. Lange) Imbach	Agaricales	Agaricaceae	LN714517.1	Slovakia
Asterophora lycoperdoides (Bull.) Ditmar	Agaricales	Lyophyllaceae	AF357037.2	Netherlands, Utrecht
Asterophora lycoperdoides (Bull.) Ditmar	Agaricales	Lyophyllaceae	AF357037.2	Netherlands, Utrecht
Asterophora parasitica (Bull.) Singer	Agaricales	Lyophyllaceae	AF357038.2	Netherlands, Utrecht
Asterophora parasitica (Bull.) Singer	Agaricales	Lyophyllaceae	AF357038.2	Netherlands, Utrecht
Calocybe aurantiaca X.D. Yu & J.J. Li	Agaricales	Lyophyllaceae	KU528828.1	China
Calocybe aurantiaca X.D. Yu & Jia J. Li	Agaricales	Lyophyllaceae	KU528828.1	China
Calocybe aurantiaca X.D. Yu & Jia J. Li	Agaricales	Lyophyllaceae	NR_156304.1	China
Calocybe badiofloccosa J.Z. Xu & Yu Li	Agaricales	Lyophyllaceae	SF593738	China
Calocybe buxea (Maire) Raithelh.	Agaricales	Lyophyllaceae	KP885633.1	Italy, Ragusa
Calocybe chrysenteron (Bull.) Singer	Agaricales	Lyophyllaceae	KP885639.1	Germany, Allenbach
Calocybe chrysenteron var. cerina (Pers.) Arnolds	Agaricales	Lyophyllaceae	KP885640.1	Germany, Ebermannstadt
Calocybe coacta J.Z. Xu & Yu Li	Agaricales	Lyophyllaceae	OK649907.1	China
Calocybe convexa X.D. Yu & Jia J. Li	Agaricales	Lyophyllaceae	KU528826.1	China
Calocybe decolorata X.D. Yu & Jia J. Li	Agaricales	Lyophyllaceae	KU528824.1	China

Calocybe decolorata X.D. Yu & Jia J. Li	Agaricales	Lyophyllaceae	KU528825.1	China
Calocybe erminea J. Z. Xu & Yu Li	Agaricales	Lyophyllaceae	MN172331.1	China
Calocybe favrei (R. Haller Aar. & R. Haller Suhr) Bon	Agaricales	Lyophyllaceae	AF357034.2	Poland, Warsaw
Calocybe favrei (R. Haller Aar. & R. Haller Suhr) Bon	Agaricales	Lyophyllaceae	AF357035.2	Poland, Warsaw
Calocybe favrei (R. Haller Aar. & R. Haller Suhr) Bon	Agaricales	Lyophyllaceae	EF421102.1	NA
Calocybe fulvipes J.Z. Xu & Yu Li	Agaricales	Lyophyllaceae	OK649910.1	China
Calocybe fulvipes J.Z. Xu & Yu Li	Agaricales	Lyophyllaceae	MT071590.1	China
Calocybe gambosa (Fr.) Donk	Agaricales	Lyophyllaceae	MZ159691.1	UK, Stafford
Calocybe gambosa (Fr.) Donk	Agaricales	Lyophyllaceae	AF357027.2	Denmark, Copenhagen
Calocybe gambosa (Fr.) Donk	Agaricales	Lyophyllaceae	MZ144137.1	NA
Calocybe gambosa f. graveolens (Pers.) Kalamees	Agaricales	Lyophyllaceae	KP192590.1	Francia
Calocybe gangraenosa (Fr.) V. Hofst., Moncalvo, Redhead & Vilgalys	Agaricales	Lyophyllaceae	AF357032.2	Poland, Warsaw
Calocybe ionides (Bull.) Donk	Agaricales	Lyophyllaceae	AF357029.2	Poland, Warsaw
Calocybe ionides (Bull.) Donk	Agaricales	Lyophyllaceae	AF357029.2	Poland, Warsaw
Calocybe lilacea X.D. Yu, Y. Zhou & W.Q. Qin	Agaricales	Lyophyllaceae	OM203538.1	China
Calocybe lilacea X.D. Yu, Y. Zhou & W.Q. Qin	Agaricales	Lyophyllaceae	OM203539.1	China
Calocybe longisterigma X.D. Yu, Y. Zhou & W.Q. Qin	Agaricales	Lyophyllaceae	OM203542.1	China
Calocybe longisterigma X.D. Yu, Y. Zhou & W.Q. Qin	Agaricales	Lyophyllaceae	OM203543.1	China
Calocybe naucoria (Murrill) Singer	Agaricales	Lyophyllaceae	KP885642.1	Italy, Predazzo
Calocybe naucoria (Murrill) Singer	Agaricales	Lyophyllaceae	AF357030.2	Poland, Warsaw
Calocybe ochracea (R. Haller Aar.) Bon	Agaricales	Lyophyllaceae	AF357033.2	Poland, Warsaw
Calocybe onychina (Fr.) Donk	Agaricales	Lyophyllaceae	MW084664.1	USA, Deschutes National Forest
Calocybe onychina (Fr.) Donk	Agaricales	Lyophyllaceae	KP885644.1	Italy, Celico
Calocybe persicolor (Fr.) Singer	Agaricales	Lyophyllaceae	AF357026.2	Poland, Warsaw
Calocybe sp. JX-2020a isolate HMJU 382	Agaricales	Lyophyllaceae	MT080028.1	NA
Calocybe subochraceus X.D. Yu, Y. Zhou & W.Q. Qin	Agaricales	Lyophyllaceae	OM203540.1	China

Calocybe subochraceus X.D. Yu, Y. Zhou & W.Q. Qin	Agaricales	Lyophyllaceae	OM203541.1	China
Calocybe vinacea J.Z. Xu & Yu Li	Agaricales	Lyophyllaceae	OK649908.1	China
Calocybe vinacea J.Z. Xu & Yu Li	Agaricales	Lyophyllaceae	OK649909.1	China
Calocybella pudica (Bon & Contu) Vizzini, Consiglio & Setti	Agaricales	Lyophyllaceae	KP858000.1	Italy, Lido di Ostia
Clitocybe nebularis (Batsch) P. Kumm.	Agaricales	Clitocybaceae	AF357063.2	Netherlands, Utrecht
Collybia nuda (Bull.) Z.M. He & Zhu L. Yang	Agaricales	Clitocybaceae	AF357062.2	USA, Durham
Entoloma abortivum (Berk. & M.A. Curtis) Donk	Agaricales	Entolomataceae	AF357019.2	Netherlands, Utrecht
Entoloma sericeum Quél.	Agaricales	Entolomataceae	AF357020.2	Netherlands, Utrecht
Entoloma sericeum Quél.	Agaricales	Entolomataceae	AF357021.2	Netherlands, Utrecht
*Gerhardtia borealis (*Fr.) Contu & A. Ortega	Agaricales	Lyophyllaceae	KP858004.1	Italy, Bellamonte
Lulesia fallax (Quél.) T.J. Baroni, Niveiro & B.E. Lechner	Agaricales	Lyophyllaceae	AF357017.2	Netherlands, Utrecht
Lulesia fallax (Quél.) T.J. Baroni, Niveiro & B.E. Lechner	Agaricales	Lyophyllaceae	AF357018.2	Netherlands, Utrecht
Lyophyllum caerulescens Clémençon ex Kibby	Agaricales	Lyophyllaceae	AF357052.2	Poland, Warsaw
Lyophyllum decastes (Fr.) Singer	Agaricales	Lyophyllaceae	AF357059.2	Poland, Warsaw
Lyophyllum decastes (Fr.) Singer	Agaricales	Lyophyllaceae	AF357060.2	Poland, Warsaw
Lyophyllum leucophaeatum (P. Karst.) P. Karst.	Agaricales	Lyophyllaceae	AF357032.2	Poland, Warsaw
Lyophyllum semitale (Fr.) Kühner	Agaricales	Lyophyllaceae	HM572552.1	Sweden
Lyophyllum semitale (Fr.) Kühner	Agaricales	Lyophyllaceae	AF357048.2	Netherlands, Utrecht
Lyophyllum semitale (Fr.) Kühner	Agaricales	Lyophyllaceae	AF357049.2	Poland, Warsaw
Lyophyllum sykosporum Hongo & Clémençon	Agaricales	Lyophyllaceae	AF357050.2	Netherlands, Utrecht
Lyophyllum sykosporum Hongo & Clémençon	Agaricales	Lyophyllaceae	AF357051.2	Poland, Warsaw
Myochromella boudieri (Khner & Romagn.) V. Hofstetter	Agaricales	Lyophyllaceae	AF357046.2	Poland, Warsaw
Myochromella boudieri (Khner & Romagn.) V. Hofstetter	Agaricales	Lyophyllaceae	AF357047.2	Poland, Warsaw
Myochromella inolens (Fries) V. Hofstetter, Clemencon, Moncalvo & Redhead	Agaricales	Lyophyllaceae	AF357045.2	Netherlands, Utrecht

Ossicaulis lignatilis (Pers.) Redhead & Ginns	Agaricales	Lyophyllaceae	DQ825426.1	USA, Durham
Rugosomyces carneus (Bull.) Bon	Agaricales	Lyophyllaceae	AF357028.2	Netherlands, Utrecht
Rugosomyces carneus (Bull.) Bon	Agaricales	Lyophyllaceae	AF357028.2	Netherlands, Utrecht
Rugosomyces obscurissimus (A. Pearson) Bon	Agaricales	Lyophyllaceae	AF357031.2	Poland, Warsaw
Sagaranella gibberosa (Jul. Schffer) V. Hofstetter, Clemencon, Moncalvo & Redhead	Agaricales	Lyophyllaceae	AF357041.2	Netherlands, Utrecht
Sagaranella gibberosa (Jul. Schffer) V. Hofstetter, Clemencon, Moncalvo & Redhead	Agaricales	Lyophyllaceae	AF357042.2	Netherlands, Utrecht
Sagaranella tylicolor (Fries) V. Hofstetter, Clemencon, Moncalvo & Redhead	Agaricales	Lyophyllaceae	AF357039.2	Poland, Warsaw
Sagaranella tylicolor (Fries) V. Hofstetter, Clemencon, Moncalvo & Redhead	Agaricales	Lyophyllaceae	AF357040.2	Poland, Warsaw
Sphagnurus paluster (Peck) Redhead & V. Hofstetter	Agaricales	Lyophyllaceae	AF357043.2	Netherlands, Utrecht
Sphagnurus paluster (Peck) Redhead & V. Hofstetter	Agaricales	Lyophyllaceae	AF357044.2	Netherlands, Utrecht
Tephrocybe ambusta (Fr.) Donk	Agaricales	Lyophyllaceae	AF357057.2	Netherlands, Utrecht
Tephrocybe ambusta (Fr.) Donk	Agaricales	Lyophyllaceae	AF357058.2	Netherlands, Utrecht
Tephrocybe anthracophila (Lasch) P.D. Orton	Agaricales	Lyophyllaceae	AF357054.2	Poland, Warsaw
Tephrocybe anthracophila (Lasch) P.D. Orton	Agaricales	Lyophyllaceae	AF357055.2	Poland, Warsaw
Tephrocybe anthracophila (Lasch) P.D. Orton	Agaricales	Lyophyllaceae	AF357056.2	Netherlands, Utrecht
Tephrocybe atrata (Fr.) Donk	Agaricales	Lyophyllaceae	AF357053.2	Netherlands, Utrecht
Tephrocybe rancida (Fr.) Donk	Agaricales	Lyophyllaceae	AF357025.2	Netherlands, Utrecht
Termitomyces heimii Natarajan	Agaricales	Lyophyllaceae	AF357022.2	USA, Durham
Termitomyces microcarpus (Berk. & Broome) R. Heim	Agaricales	Lyophyllaceae	AF357023.2	USA, Durham
Termitomyces subhyalinus Moncalvo, Vilgalys, Redhead, J.E. Johnson, T.W. James, Aime, Hofstetter, Verduin, E. Larsson, Baroni, R.G. Thorn, Jacobsson, Clémençon & Miller	Agaricales	Lyophyllaceae	AF357024.2	Poland, Warsaw

Tricholoma pardinum (Pers.) Quél.	Agaricales	Tricholomataceae	AF357014.2	NA
Tricholoma portentosum (Fr.) Quél.	Agaricales	Tricholomataceae	AF357015.2	NA
Tricholoma subaureum Ovrebo	Agaricales	Tricholomataceae	AF357016.2	NA
Tricholomella constricta (Fr.) Zerova ex Kalamees	Agaricales	Lyophyllaceae	AF357036.2	Poland, Warsaw
Tricholomella constricta (Fr.) Zerova ex Kalamees	Agaricales	Lyophyllaceae	DQ825429.1	NA

Note: NA = not available. [a] = natural fruiting site or herbarium
